# High-efficiency fungal pathogen intervention for seed protection: new utility of long-chain alkyl gallates as heat-sensitizing agents

**DOI:** 10.3389/ffunb.2023.1172893

**Published:** 2023-07-28

**Authors:** Jong H. Kim, Kathleen L. Chan, William M. Hart-Cooper, Jeffrey D. Palumbo, William J. Orts

**Affiliations:** ^1^Foodborne Toxin Detection and Prevention Research Unit, Western Regional Research Center, Agricultural Research Service, United States Department of Agriculture (USDA-ARS), Albany, CA, United States; ^2^Bioproducts Research Unit, Western Regional Research Center, Agricultural Research Service, United States Department of Agriculture (USDA-ARS), Albany, CA, United States

**Keywords:** alkyl gallates, bacteria, fungi, heat sensitization, hurdle technology, seed protection, synergism, thermal resistance

## Abstract

Control of food-contaminating fungi, especially pathogens that produce mycotoxins, is problematic since effective method for intervening fungal infection on food crops is often limited. Generally Regarded As Safe (GRAS) chemicals, such as natural compounds or their structural derivatives, can be developed as antimicrobial agents for sustainable food/crop production. This study identified that long-chain alkyl gallates, i.e., octyl-, nonyl-, and decyl gallates (OG (octyl 3,4,5-trihydroxybenzoic acid), NG, DG), can function as heat-sensitizing agents that effectively prevent fungal contamination. Out of twenty-eight candidate compounds and six conventional antifungal agents examined, the heat-sensitizing capacity was unique to the long-chain alkyl gallates, where OG exhibited the highest activity, followed by DG and NG. Since OG is a GRAS compound classified by the United States Food and Drug Administration (FDA), further *in vitro* antifungal studies were performed using OG. When OG and mild heat (57.5°C) were co-administered for 90 seconds, the treatment achieved > 99.999% fungal death (> 5 log reduction). Application of either treatment alone was significantly less effective at reducing fungal survival. Of note, co-application of OG (3 mM) and mild heat (50°C) for 20 minutes completely prevented the survival of aflatoxigenic *Aspergillus flavus* contaminating crop seeds (*Brassica rapa* Pekinensis), while seed germination rate was unaffected. Heat-sensitization was also determined in selected bacterial strains (*Escherichia coli*, *Agrobacterium tumefaciens*). Altogether, OG is an effective heat-sensitizing agent for control of microbial pathogens. OG-mediated heat sensitization will improve the efficacy of antimicrobial practices, achieving safe, rapid, and cost-effective pathogen control in agriculture/food industry settings.

## 1 Introduction

Chemical seed treatment is one of the crop protection strategies which enables healthy crop establishment in the fields through better seed preservation/germination and plant protection against pathogens. Compared to other protection strategies, such as foliar/furrow spraying during crop season, seed treatment can achieve a low-cost crop protection since the method requires a relatively low amount of active ingredients (Lamichhane et al., 2020). Chemical seed treatment also provides disease control at the earlier growth stage of crops. It has been estimated that fungi cause around 20% of world crop/food losses (Fisher et al., 2018). Considering the limited efficacy of conventional seed sanitation methods (Ali et al., 2021), directly affecting food safety and food security, the development of safe, novel alternatives to the toxic chemical seed treatments is highly needed.

From a redox-chemistry perspective, the generation of reactive oxygen species (ROS) is the contributing mechanism of toxicity to crops under either biotic (pathogen attack) or abiotic (heat, drought, etc.) stresses (Kurek et al., 2019; Mohammadi et al., 2021). ROS accumulation by biotic/abiotic stresses results in the degradation or deterioration of cellular components, such as membrane phospholipids, proteins and genetic materials (Jeevan Kumar et al., 2015; Ratajczak et al., 2015; Mohammadi et al., 2021). Therefore, oxidative damage in crop seeds *via* biotic/abiotic stresses causes reduced seed germination rate as well as loss of seed viability, leading to crop losses.

Meanwhile, selected natural products and their structural derivatives, functioning as both antioxidants and prooxidants, can modulate cellular redox status, and hence significantly impact cellular redox homeostasis in the hosts or infecting pathogens (Jacob, 2014). For example, the antioxidant activity of redox-active compounds can scavenge toxic ROS in the host cells, minimizing oxidative damage in seeds during storage (Macedo et al., 2018). Conversely, the prooxidant activity of redox-active molecules negatively affects pathogens by disrupting redox-sensitive structures in microbes, thus effectively preventing pathogen growth (Jacob, 2006). Therefore, the dual function, namely antioxidant and prooxidant potential, of certain redox-active molecules can be the key element for the development of new, sustainable seed-protection products.

Heat treatment is also one of the important control strategies for preventing pathogen contamination in agricultural or food production (Dagnas and Membré, 2013; Salazar-Orbea et al., 2021; Soni et al., 2022; Yao et al., 2023). Artificially applied heat negatively affects the survival of microbial pathogens, which can ensure the reduction of pathogen contamination or postharvest decay. For example, Hawkins et al. (2005) showed that heat treatment at 70°C significantly reduced *A. flavus* infection of maize kernels, thereby reducing postharvest AF contamination. However, intensive heat treatment often results in deterioration of the quality of the crop seeds or food products, such as seed integrity/viability, nutritional value, texture, etc. (Kim et al., 2015a; Sánchez-Bravo et al., 2022). High heat treatment (steaming, roasting, blanching, etc.) during pasteurization is also expensive. Therefore, there is an increased demand for the development of new, alternative heat treatment strategies that contribute to early pathogen intervention and toxin control, as well as the quality of harvested crops (Masotti et al., 2021). The development of pathogen resistance to thermal treatments is also an increasing public health concern, especially for the food safety sectors (Ros-Chumillas et al., 2015; Peng et al., 2017; Knowles et al., 2020; Santos et al., 2020; Timmermans et al., 2020).

Hurdle technology is an approach where combined application of different types of preservation method at much reduced individual intensities could achieve increased effectiveness of antimicrobial intervention (Leistner, 2000; Aaliya et al., 2021; Soni and Brightwell, 2022). Examples of heat-based hurdle technology include: (1) reduction of *Escherichia coli* and *Saccharomyces cerevisiae* (9.0 and 8.0 decimal reductions, respectively) in orange juice *via* pulsed electric field (PEF) and thermal treatments (Lee et al., 2021); (2) reduction of thermal stability of PEF-treated *Bacillus cereus* spores at initial temperatures of 80 and 75°C (Soni et al., 2020); (3) enhancement of the microbial safety of fresh-cut bell pepper by inactivating *Listeria monocytogenes* and *Salmonella enterica* serovar *Typhimurium* with combined treatment of acidic electrolyzed water, ultrasound and mild heat (60°C) (Luo and Oh, 2016); (4) inactivation of *E. coli* O157:H7 on fresh-cut spinach by optimizing low-temperature (45–65°C) blanching, combined with calcium treatment (calcium hydroxide) (Kim et al., 2015b); (5) inactivation of *S. enterica* serovar Montevideo on mung bean seeds by combined treatment of dry heat (55–70°C) and chlorine dioxide gas (Annous and Burke, 2015); and (6) significant decrease in the heat resistance of *Clostridium sporogenes* by combined treatment of electro-activated solutions and heat (Liato et al., 2015). However, in many hurdle-technology approaches combining chemicals and heat, the co-applied chemicals were synthetic additives, which are not preferred by consumers.

In this study, we investigated a new hurdle technology by examining the mild heat (50–57.5°C)-sensitizing capability of thirty-four compounds, currently used as food additives or antifungal agents, for the elimination of mycotoxin-producing *A. flavus* contaminated on the surfaces of crop seeds.

## 2 Methods

### 2.1 Microorganisms

Microbial strains used in this study are described in **Supplementary Table S1**. The species of *Aspergillus* and *Penicillium* were cultured at 35°C and 28°C, respectively, on potato dextrose agar (PDA) (Becton, Dickinson and Co., NJ, USA). *E. coli* and *Agrobacterium tumefaciens* were grown at 37°C and 28°C, respectively, on Luria Broth (LB) (Becton, Dickinson and Co., NJ, USA) agar. The pH of the media was adjusted to pH 7.0.

### 2.2 Chemicals

Chemicals used in this study were procured from Sigma-Aldrich Co. (St. Louis, MO, USA) as follows: (1) Aromatic compounds currently used as food additives (benzaldehyde, benzoic acid, carvacrol, cinnamaldehyde, cinnamic acid, 2,4-dihydroxybenzoic acid, 3,4-dihydroxybenzoic acid, guaiacol, 2-hydroxy-4-methoxybenzaldehyde, 2-hydroxy-4-methylbenzaldehyde, methyl benzoic acid, salicylaldehyde, salicylic acid, thymol, vanillin, vanillic acid, vanillylacetone) (U.S. Food and Drug Administration (FDA), 2022), (2) Gallate and its alkyl derivatives [short chain alkyl gallates: methyl-, ethyl-, propyl-, butyl gallate; long chain alkyl gallates: octyl-, nonyl- and decyl gallate (**Supplementary Figure S1A**)], (3) Antimycotic drugs/fungicides (caspofungin, cyprodinil, fludioxonil, itraconazole, pyraclostrobin, pyrimethanil), and (4) Other food additives/antimicrobial compounds (hydrogen peroxide, sodium lauryl sulfate, 2,3-dihydroxybenzaldehyde). Each compound was dissolved in dimethyl sulfoxide (DMSO; final DMSO concentration applied: < 1% in solution) except hydrogen peroxide, which was diluted in distilled water, before incorporation into culture medium or phosphate-buffered saline (PBS). In all tests, control plates (No treatment) contained DMSO at levels equivalent to that of cohorts receiving antifungal agents, within the same set of experiments.

### 2.3 Antimicrobial bioassay

To determine the optimum temperature and time duration for screening heat sensitizers, fungal (*A. flavus* strain NRRL3357) survivability was tested by exposing fungal spores (1.5 × 10^5^ to 2.1 × 10^5^ CFU/mL) to five different temperatures (50, 55, 57.5, 60, 65°C) at various time points (0.5, 1.0, 1.5, 2.0, 2.5, 3.0, 3.5, 4.0, 4.5, 5.0 min), where the 90 seconds (1.5 min) treatment with mild heat (50, 55, 57.5°C) resulted in the survival/growth of fungal spores (Levels of fungal survival: 50 > 55 > 57.5°C, high to low; data not shown). Therefore, “57.5°C + 90 seconds” setting was chosen for screening heat-sensitizing compounds (see **Figure S1B** and below).

The heat-sensitizing effect of twenty-eight compounds (aromatic compounds, alkyl gallates, other food additives) (Test concentrations: 0.025, 0.050, 0.075, 0.100, 0.200, 0.300, 0.400, 0.500, 0.600 or 0.700 mM) and six drugs/fungicides (Test concentrations: 1, 2, 4, 8, 16, 32, 64 ppm) (**Table 1**) were examined using *A. flavus* strain NRRL3357. Two hundred microliters of fungal spores (1.5 × 10^5^ to 2.1 × 10^5^ CFU/mL) dispersed in PBS were transferred to individual microfuge tubes (1.5 mL volume), where (1) “control” tube contained DMSO only, while (2) “treatment” tube contained test chemical/drug at the respective concentration. The tubes were then treated with mild heat (57.5°C) or maintained at room temperature (RT, 22.0°C) for 90 seconds. After treatments, entire volume (200 µL) of a sample from each microfuge tube was spread onto fresh recovery PDA, and plates were incubated at the optimum temperatures for each strain. Fungal survival was monitored *via* colony counts after 24 to 72 h of incubation.

**Table 1:** MFC of prospective heat-sensitizing agents (Determination of > 5 log fungal reduction with mild heat co-treatment). Note that OG possesses the highest antifungal activity at either temperature.

Temperature	MFC at 22.0°C	MFC at 57.5°C
Compounds		
Phenolic derivatives (μM)		
Benzaldehyde	> 700	> 700
Benzoic acid	> 700	> 700
Carvacrol	> 700	> 700
Cinnamaldehyde	> 700	> 700
Cinnamic acid	> 700	> 700
2,4-Dihydroxybenzoic acid	> 700	> 700
3,4-Dihydroxybenzoic acid	> 700	> 700
Guaiacol	> 700	> 700
2-Hydroxy-4-methoxybenzaldehyde	> 700	> 700
2-Hydroxy-4-methylbenzaldehyde	> 700	> 700
Methyl benzoic acid	> 700	> 700
Salicylaldehyde	> 700	> 700
Salicylic acid	> 700	> 700
Thymol	> 700	> 700
Vanillin	> 700	> 700
Vanillic acid	> 700	> 700
Vanillylacetone	> 700	> 700
Alkyl gallates (μM)		
Gallate	> 700	> 700
Methyl gallate	> 700	> 700
Ethyl gallate	> 700	> 700
Propyl gallate	> 700	> 700
Butyl gallate	> 700	> 700
Octyl gallate	250	180*
Nonyl gallate	> 700	400*
Decyl gallate	> 700	200*
Antimycotic drugs/fungicides (μg/mL; ppm)		
Caspofungin	> 64	> 64
Cyprodinil	> 64	> 64
Fludioxonil	> 64	> 64
Itraconazole	64	> 64
Pyraclostrobin	> 64	> 64
Pyrimethanil	> 64	> 64
Other food additives (μM)		
Hydrogen peroxide	> 700	> 700
Sodium lauryl sulfate	> 700	> 700
2,3-Dihydroxybenzaldehyde	> 700	> 700

* P = 0.155 for long-chain alkyl gallates.

Then, the most potent heat-sensitizing agent, octyl gallate (OG), was selected, and was examined for further heat-sensitization analyses in other microorganisms (Fungi: *Aspergillus* and *Penicillium* species; bacteria: *E. coli*, *A. tumefaciens*) (Test concentrations: 0.05, 0.075, 0.10, 0.11, 0.12, 0.13, 0.14, 0.15, 0.16, 0.17, 0.18, 0.19, 0.20, 0.25, 0.30 mM) (See **Table 2** and **Table S2**). Bacterial cell suspensions were prepared from overnight cultures on LB agar and suspended in PBS at a density of approximately 1 × 10^8^ CFU/mL. As described in the *A. flavus* strain NRRL3357 test (see above), spore or cell suspensions were treated with mild heat (57.5°C) or maintained at RT (22.0°C) (90 seconds). After heat treatments, entire volume (200 µL) from each microfuge tube was spread onto fresh LB agar (for bacteria) or PDA (for fungi), and plates were incubated at the optimum temperature for the respective microorganisms (i.e., 35°C for *Aspergillus*, 37°C for *E. coli*, and 28°C for *Penicillium*/*A. tumefaciens*, respectively). The survival of test microorganisms was monitored *via* colony counts after 24 to 72 h of incubation.

**Table 2:** Heat-sensitizing capability of OG in different fungi.

Treatment	MFC of OG (µM), 22.0°C	MFC of OG (µM), 57.5°C
Fungi		
*A. flavus* NRRL3357	250	180
*A. flavus* NRRL4212	> 300	180
*A. parasiticus* NRRL2999	> 300	200
*A. parasiticus* NRRL5862	> 300	300
*A. brasiliensis* ATCC16404	> 300	160
*P. expansum* W1	> 300	160
*P. expansum* FR2	> 300	190
*P. expansum* W2	> 300	170
*P. expansum* FR3	> 300	160
*P. italicum* 983	200	100
*P. griseofulvum* 2159	> 300	180
*P. chrysogenum* 824	250	130
**Average**	> 283.3	175.8 (*p* < 0.005)

### 2.4 Antifungal seed disinfection bioassays using OG

Effect of OG plus mild heat (hurdle technology) on the extent of seed sanitation (*Brassica rapa* Pekinensis; Chinese cabbage hybrid) (Plant nursery, Oakland, CA, USA) was investigated. Twelve cabbage seeds, unsterilized, were transferred to each 1.5 mL microcentrifuge tube filled with 1 mL (final volume) of distilled water, containing 0, 2 mM, 3 mM or 4 mM of OG, without or with *A. flavus* (1 × 10^5^ CFU/mL), and held at 20°C or 50°C for 20 or 30 min. After each treatment, the treated seeds (Total 12 seeds/tube) were transferred to PDA and were incubated in the dark at RT (22.0°C) for seed germination. The growth of seedlings and the level of fungal contamination on the surfaces of germinated seeds were monitored for up to 7 days.

### 2.5 Statistical analysis

Statistical analysis (student’s *t*-test) was performed according to “Statistics to use” (Kirkman, 1996) where *p* < 0.05 was considered significant.

## 3 Results

### 3.1 Selection of long-chain alkyl gallates (octyl-, nonyl-, decyl gallate; OG, NG, DG) as heat-sensitizing agents

Heat-sensitizing capacity of test compounds (Twenty-eight food additives/structural derivatives and six conventional drugs/fungicides; see **Methods**) was investigated by determining minimum fungicidal concentration (MFC; achieving > 5 log reduction) of each compound at room temperature (RT) (22.0°C; Control) or at mild heat (57.5°C) in *A. flavus* strain NRRL3357. As shown in **Table 1**, *A. flavus* strain NRRL3357 survived (w/less than 5 log reduction) at either RT or 57.5°C with the co-treatment of most compounds examined at up to 700 µM (aromatic compounds, short-chain alkyl gallates (gallate, methyl-, ethyl-, propyl-, butyl gallate), other food additives, or 64 ppm of conventional drugs/fungicides).

On the other hand, when fungi were co-treated with as low as 180, 400 or 200 µM of long-chain alkyl gallates (OG, NG or DG, respectively), at 57.5°C, > 5 log fungal reduction was achieved. Results indicate that long-chain alkyl gallates possess potent heat-sensitizing capability, where OG exhibited the highest activity by showing the lowest MFC (180 µM) (**Figure S2A**), followed by DG (MFC: 200 µM) and NG (MFC: 400 µM) (*P* = 0.155) (**Table 1**). Of note, OG alone could also achieve > 5 log fungal reduction at RT (at as low as 250 µM), while NG or DG did not exert similar level of antifungal activity at the same condition even with the highest concentration (700 µM) applied (**Table 1**), indicating the high potential of OG as an effective antifungal agent.

### 3.2 Heat-sensitizing activity of OG in other fungi

Heat-sensitizing activity of OG was then investigated in other fungal pathogens/contaminants (five *Aspergillus* and seven *Penicillium* strains) including fungicide resistant strains (*P. expansum* FR2 and FR3 showing resistance to fludioxonil, a phenylpyrrole fungicide) (See **Table S1**). As shown in **Table 2**, co-application of OG with mild heat (57.5°C) significantly (*P* < 0.005) lowered MFC values of OG in all fungal strains examined. The average MFC of OG determined in twelve fungi was decreased from > 283.3 µM to 177.5 µM, when temperature was increased from 22.0°C (RT) to 57.5°C (mild heat). Therefore, results indicated that OG possesses a heat-sensitizing capability in various fungal pathogens contaminating food crops. While not shown in **Table 2**, modest level of heat sensitization was also achieved in the heat-resistant environmental fungus *Neosartorya fischeri* (ATCC 96468), where > 5 log fungal reduction was observed with 150 or 120 µM of OG at 22.0°C (RT) or 57.5°C, respectively (Data not shown). We also determined the heat-sensitizing activity of OG in bacteria (*A. tumefaciens*, *E. coli*) (**Table S2**; **Figure S2B**). Therefore, results further indicated that OG possesses heat-sensitizing capability in broad-spectrum microbial pathogens or contaminants.

### 3.3 Indifferent interactions between the conventional antifungal drugs/fungicides and mild heat treatment

In parallel, heat-sensitizing capability of conventional antifungal drugs or fungicides was investigated in *A. flavus* strain NRRL3357, where their efficacy as heat sensitizers was compared to OG (See above). As shown in the **Supplementary Figure S3**, co-application of any of the six drugs/fungicides with mild heat (57.5°C) did not achieve 5 log fungal reduction, even at the highest concentration tested (64 ppm). For example, mild heat (57.5°C) plus caspofungin (cell wall-disrupting drug; up to 64 ppm; 90 sec) allowed the full growth/survival of fungi (**Figure S3**). Mild heat (57.5°C) plus itraconazole (cell membrane-interfering drug; up to 64 ppm; 90 sec) actually resulted in loss of drug activity by heat, rendering the survival (or slightly better growth compared to RT) of fungi (**Figure S3**). Therefore, these six conventional drugs or fungicides tested did not possess heat-sensitizing capability.

### 3.4 Seed disinfection *via* heat-sensitizing activity of OG

**Figure 1:**
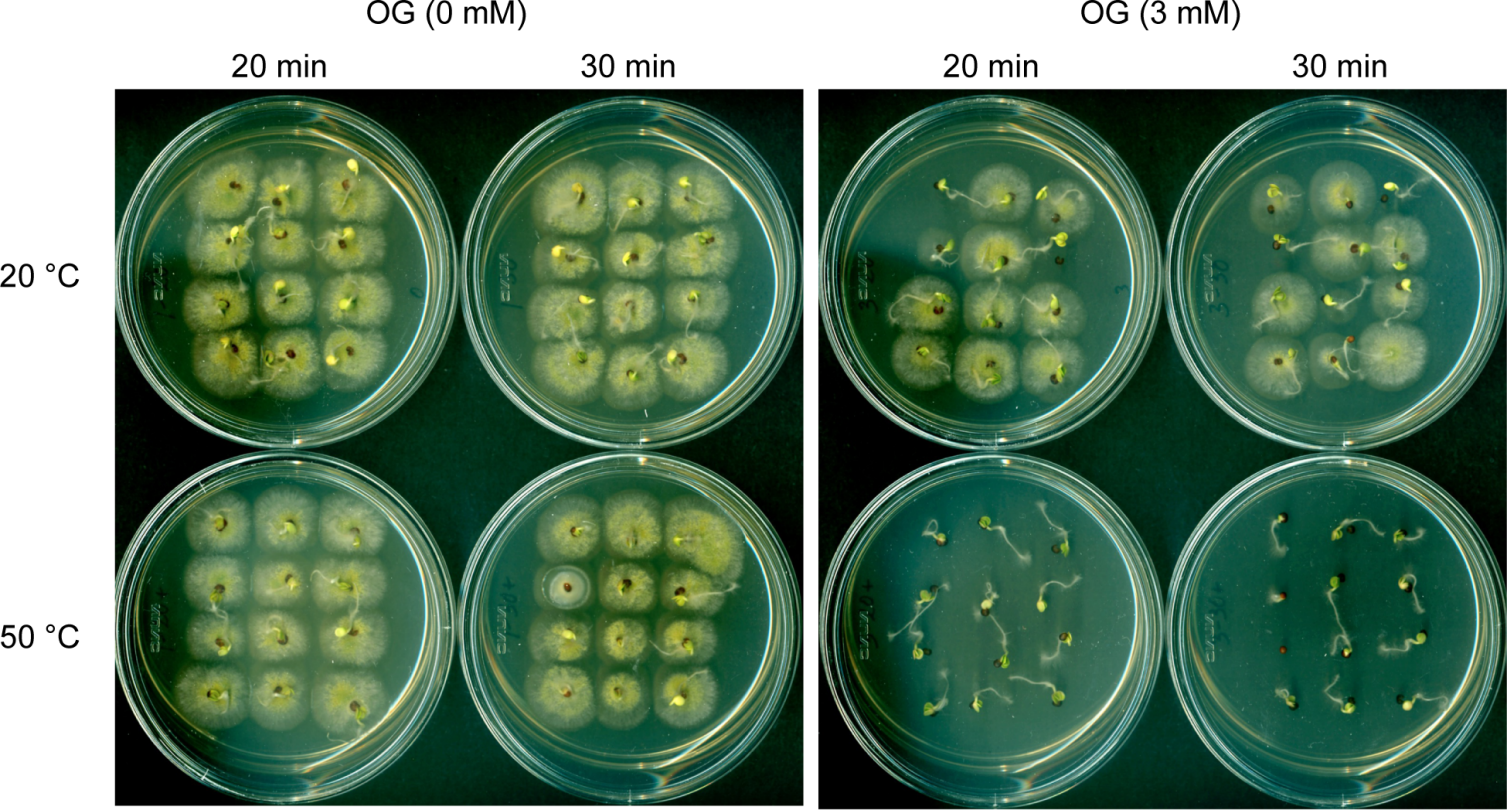
Effect of OG plus mild heat co-treatment (hurdle technology) on the disinfection of cabbage seeds inoculated with *A. flavus* NRRL3357.

Finally, heat-sensitizing capacity of OG was investigated further in the crop seeds (*Brassica rapa* Pekinensis; Chinese cabbage hybrid), which were artificially contaminated with *A. flavus* NRRL3357 spores. OG (0, 2, 3, 4 mM) and mild heat (50°C; Control: 20°C) were co-administered for 20 or 30 minutes, respectively, and the seed germination rate as well as the level of fungal contamination was determined. As shown in **Table 3** (See also **Figure 1**), co-treatment of OG at as low as 3 mM and mild heat (50°C) for 20 minutes completely inhibited the growth of *A. flavus* on the surfaces of the seeds, while the germination rate of the crop seeds was not affected when compared to the control. Whereas, at 2 mM of OG, low level of fungal contamination still occurred (in both 20- and 30-minute treatments at 50°C); slight decrease in seed germination rate was also determined at 4 mM of OG (at 50°C).

**Table 3:** Effect of OG plus mild heat treatment (hurdle technology) on sanitation of cabbage seeds contaminated with *A. flavus* NRRL3357.

OG (mM)	Temp. (°C)	Time (Minutes)	No. of seedlings germinated	No. of seedlings infected	No. of seedlings naturally contaminated ^c^
0 (w/o *A. flavus*)	20	20	12	0	1
		30	12	0	0
	50	20	12	0	0
		30	12	0	2
% Seed germination			100 ± 0	–	
% *A. flavus* infection			–	N/A ^e^	
0	20	20	12	12	0
		30	12	12	0
	50	20	12	12	0
		30	12	12	1
% Seed germination			100 ± 0	–	
% *A. flavus* infection			–	100 ± 0	
2	20	20	12	12	0
		30	12	10	1
	50	20	12	4	0
		30	10	1	0
% Seed germination			96 ± 8 (^a^ *P* = 0.356)	–	
% *A. flavus* infection			–	57 ± 43 (^b^ *P* = 0.086)	
3	20	20	12	10	0
		30	12	10	0
	50	20	12	0 ^d^	0
		30	11	0 ^d^	0
% Seed germination			98 ± 4 (^a^ *P* = 0.356)	–	
% *A. flavus* infection			–	42 ± 48 (^b^ *P* = 0.052)	
4	20	20	12	12	0
		30	12	11	0
	50	20	11	0 ^d^	0
		30	11	0 ^d^	0
% Seed germination			96 ± 5 (^a^ *P* = 0.134)	–	
% *A. flavus* infection			–	48 ± 56 (^b^ *P* = 0.109)	

a Student’s *t*-test for paired data (i.e., % seed germination under OG treatment) was vs. mean % seed germination without OG treatment.

b Student’s *t*-test for paired data (i.e., % *A. flavus* infection under OG treatment) was vs. mean % *A. flavus* infection without OG treatment.

c Number of seedlings with natural contamination identified (before *A. flavus* inoculation).

d Denotes 100% *A. flavus* death.

e N/A, not applicable.

## 4 Discussion

In this study, the new utility of long-chain alkyl gallates (OG, NG, DG) as heat-sensitizing agents was identified for effective control of fungal pathogens on crop seeds. Among thirty-four compounds (including six conventional drugs/fungicides) investigated, OG exhibited the highest heat-sensitizing capability, followed by DG and NG.

OG has been classified as a GRAS compound and used as an antioxidant or a color additive in the United States (U.S. Food and Drug Administration (FDA), 2022). In previous studies, OG was shown to possess antifungal activity related to its redox properties. OG functions as a pro-oxidant in fungal cells, hence induces oxidative stress-mediated cytotoxicity in fungi (Sierra-Campos et al., 2009). OG also exerted inhibitory action on the mitochondrial alternative oxidase (AOX) (Sierra-Campos et al., 2009). When fungal pathogens are treated with inhibitors of complex III in the mitochondrial respiratory chain (MRC), such as strobilurin fungicides, the inhibition not only prevents cellular energy (ATP) generation but also triggers cellular oxidative stress, which is caused by abnormal leakage of electrons from the MRC (Fujita et al., 2004; Ruy et al., 2006). The escaped electrons then cause oxidative damage to cellular structures including cell membranes. However, the intrinsic AOX activity renders the completion of electron transfer and ATP production in fungi (Costa-de-Oliveira et al., 2012), thus overcoming the toxicity caused by complex III-inhibitors and developing fungal resistance to the fungicides (Costa-de-Oliveira et al., 2012). Conversely, the AOX-inhibitory effect of OG prevents the development of strobilurin resistance in fungi. OG also induced a morphological change (viz., switch from yeast to a mycelium) in the fungus *Ustilago maydis*, possibly by the formation of toxic lipid peroxides; this activity is unique to OG while the short-chain alkyl gallate propyl gallate causes negligible cell changes (Sierra-Campos et al., 2009). Other studies have shown that OG inhibits mitochondrial respiration in the fungus *Rhizopus stolonifera*, a causative agent for the most destructive postharvest storage diseases in fruits/vegetables (Robles-Martínez et al., 2013). Other properties that contribute to antifungal activity of OG include nonionic surfactant action, interrupting or disorganizing the lipid bilayer-protein interface in fungal cells, and inhibition of plasma membrane H^+^-ATPase (Fujita and Kubo, 2002). In addition, our previous work showed that OG destabilizes cell wall integrity as well as debilitates the fungal antioxidant system (Kim et al., 2018). Collectively, studies indicated that OG could target multiple cellular components including cellular membranes, cell walls, AOX, antioxidant system, etc. (See **Figure S4**). We reasoned that the multifaceted antifungal characteristics of OG render this compound function as a better heat-sensitizing agent against fungal pathogens compared to other compounds examined in this study.

Meanwhile, the role of heat stress as an inducer of oxidative stress has also been documented in higher organisms, such as in poultry (Lin et al., 2006; Akbarian et al., 2016). Heat stress induces expression of heat shock proteins (HSPs) HSP70 and HSP90, resulting in mitochondrial dysfunction and tissue damage (Akbarian et al., 2016). Interestingly, dysfunction of MRC also activates a HSP response in cells which is mediated by oxidative stress signaling (Barrett et al., 2004). Therefore, we surmised that the synergism between OG- and heat-triggered oxidative stresses is also one mechanism of OG-mediated heat sensitization in fungi, resulting in the increased inhibition of fungal growth after the dual treatment. For the further analysis of OG/heat oxidative stress response by *A. flavus*, the use of genetic/genomic tools, such as the forty-three *A. flavus* orthologs of *S. cerevisiae* genes involved in gene regulation, signal transduction (*SHO1*, *HOG1*) and antioxidation (*CTT1*, *CTA1, SOD2*), etc. (Kim et al., 2005) and/or Mitogen-Activated Protein Kinase (MAPK) gene knockout mutants recently available, for example *AflBck1* and *AflSlt2* mutants (Zhang et al., 2020a; Zhang et al., 2020b), will help elucidate which genes involved in oxidative stress or downstream targets of MAPK pathways are affected in *A. flavus* under OG/heat treatment.

We also speculate that there might be a crosstalk between the oxidative stress and cell wall integrity signaling systems (i.e., MAPK systems) under OG stress, and therefore, the two MAPK systems could be positively coordinated to counteract the OG stress in fungi. Of note, crosstalk between the cell wall and oxidative stress response pathways have been documented previously in different fungi (Rodríguez-Peña et al., 2010; Manfiolli et al., 2019; Mattos et al., 2020).

We observed further that, among the three long-chain alkyl gallates exerting heat sensitization, OG (C_8_) exhibited the highest activity followed by DG (C_10_) and NG (C_9_). It’s intriguing to note that, in a previous investigation (Fujita and Kubo, 2002), OG exhibited the most potent antifungal activity against the model fungus *S. cerevisiae* when compared to propyl (C_3_)- or dodecyl (C_12_) gallate. OG rapidly bound to *S. cerevisiae* cells, while the dodecyl gallate having a longer alkyl chain did not bind to the cells. It was determined that the hydrophilic moiety of OG (catechol/pyrogallol) bound to the hydrophilic portion of the surface of fungal membrane, thus functioning as a surfactant to interfere with the membrane bilayer to elicit the antifungal activity, whereas dodecyl gallate could not (Fujita and Kubo, 2002). Similar phenomenon might occur during the heat sensitization as tested against filamentous fungal pathogens in this study, where C_8_ is also the cutoff alkyl chain length for the optimum antifungal activity. Precise determination of the effect of OG on *A. flavus* membrane as well as cell wall integrity warrants future in-depth investigation.

In our study, heat sensitization by OG was also observed in bacteria (*E. coli*, *A. tumefaciens*). In the previous investigations, OG also inhibited the growth of bacterial pathogens, including the foodborne pathogen *Staphylococcus aureus* (Rúa et al., 2011) as well as *Enterococcus faecalis* (dairy isolates) which expresses different virulence factors (Gutiérrez-Fernández et al., 2013). We assume that the mechanism of antibacterial action could be similar to that identified in fungi, such as targeting the cellular antioxidant system, etc. However, since there are considerable differences between fungi and bacteria cell wall and membrane, it would be necessary to verify molecularly how OG interact with bacterial membrane.

Regarding the detoxification of mycotoxins such as aflatoxins produced by *A. flavus*, various approaches have been reported to inactivate mycotoxins, including biological, chemical and physical methods. The biocontrol-based mycotoxin detoxification is widely recognized as specific, efficient, environment-friendly and sustainable methods; for example, mycotoxin removal by probiotics, cell-binding of mycotoxins, biodegradation, microbial consortia and recombinant enzyme analysis (Kim and Chan, 2014; El-Baky and Amara, 2021; Liu et al., 2022; Maidana et al., 2022; Zadravec et al., 2022). On the other hand, information on detailed mechanisms of mycotoxin degradation by biocontrol approach is currently scarce (Zadravec et al., 2022), for which LC-MS/MS or GC-MS/MS methods may help to elucidate the pathway of mycotoxin biodegradation (Agriopoulou et al., 2020). Decontamination of mycotoxin by physical techniques includes sorting and separation, washing, solvent extraction, heating, irradiation, and adsorption (Yousefi et al., 2021; Wang et al., 2022), while that by chemical techniques primarily includes alkaline and ozone treatments (Conte et al., 2020) among other plethora of methods (El-Baky and Amara, 2021). Thus, the methods of mycotoxin detoxification are broadly available; however, the efficacy can be limited depending on how the method is used, the target, the contamination of the environment, among others. Of note, the correlation between fungicide resistance and enhanced mycotoxin production has also been documented (Kim et al., 2015a). Therefore, research is continually needed to develop control measures to effectively reduce or eliminate fungal pathogens during agricultural and food processing practices. In this respect, seed sanitation can provide an effective/economic crop protection and mycotoxin control considering the approach needs a relatively low amounts of active ingredients/fungicides for fungal control (Lamichhane et al., 2020) and also achieves disease control at the earlier growth stage of crops.

While co-application of OG at 3 mM and mild heat (50°C), 20 minutes, completely inhibited *A. flavus* contamination on the seed surfaces without affecting the seed germination frequency, the slight decrease in seed germination frequency at 4 mM of OG (at 50°C) indicates OG at higher doses might negatively affect the seed germination possibly by interrupting cellular redox homeostasis during seed germination (Nietzel et al., 2020; Albertos et al., 2021). Whereas, lower concentration (2 mM) of OG could not provide sufficient antifungal activity for control of *A. flavus*, strongly indicating optimization of OG treatment condition is necessary when OG is applied to seed treatments.

In summary, the results from this study provide novel advantages over conventional antimicrobial practices, establishing new hurdle technology, which will also alleviate side effects associated with current antimicrobial intervention methods. OG and other long-chain alkyl compounds could be used as an ingredient in seed disinfection formulations, which can serve as safe, sustainable alternatives to conventional seed-disinfecting fungicides. In addition to the antimicrobial (pro-oxidant) activity, the antioxidant activity of the redox-active OG could be useful for scavenging toxic ROS, thus minimizing the oxidative damages in seeds during storage, for example, *via* seed coating. Finally, determination of the precise mechanism of OG-mediated heat sensitization in different microbes warrants future in-depth study.

## Data availability statement

The original contributions presented in the study are included in the article/**Supplementary Material**. Further inquiries can be directed to the corresponding author.

## Author contributions

JK conceived and designed the experiments including methodology and validation. JK and KC performed experiments, formal analysis and data curation. WH-C, JP, and WO performed formal analysis and provided resources. JK wrote the original paper and KC, WH-C, JP, and WO reviewed and edited the paper. WO performed project administration. All authors have read and agreed to the published version of the manuscript. All authors contributed to the article.
