# Supplementary material for: High-efficiency fungal pathogen intervention for seed protection: new utility of long-chain alkyl gallates as heat-sensitizing agents
Source: Front Fungal Biol. 2023 Jul 28;4:1172893. doi: 10.3389/ffunb.2023.1172893 (PMC10512402; doi:10.3389/ffunb.2023.1172893)
Supplement: Supplementary file 1 [file DataSheet_1.pdf]

Supplementary Material

1.1 Supplementary Figures

A

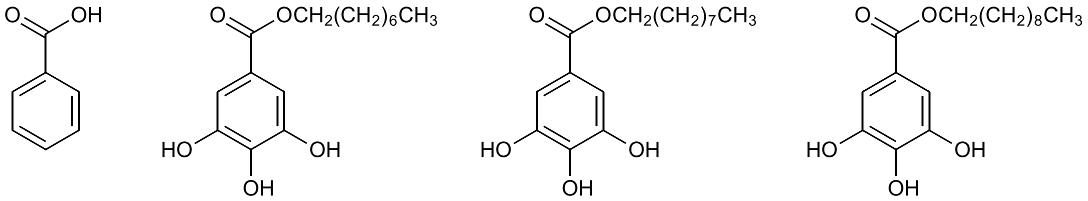

1 **2** **3** **4**

B

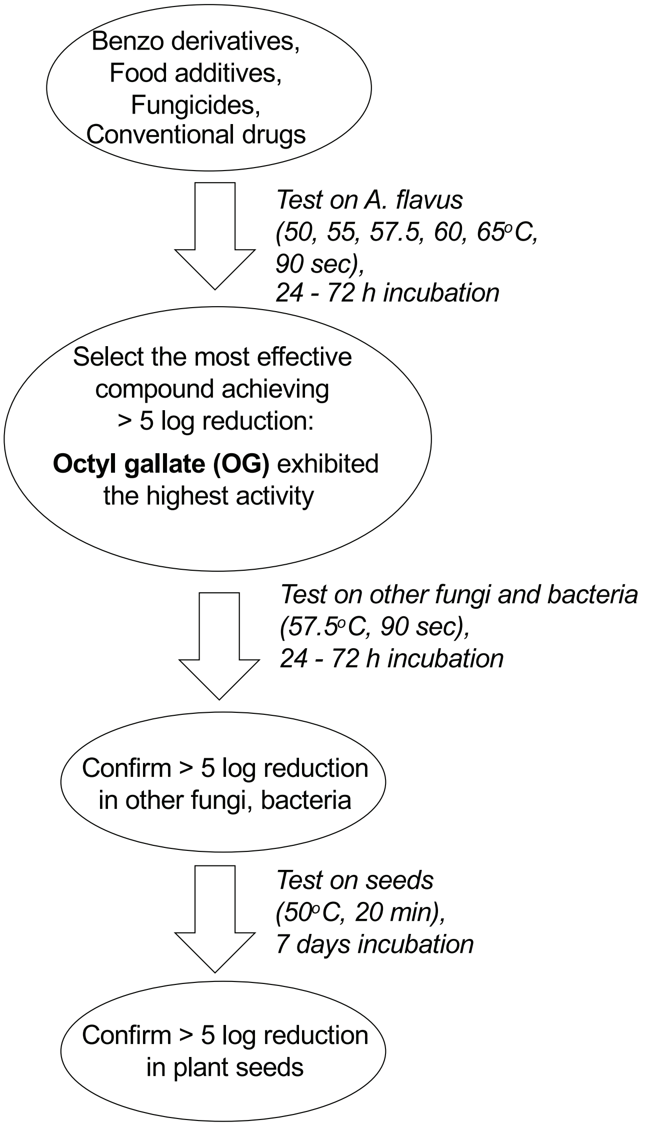

Figure S1. (A) Structure of long-chain alkyl gallates used as heat-sensitizing agent in this study. (1) Gallate base structure; (2) Octyl gallate (OG); (3) Nonyl gallate (NG); (4) Decyl gallate (DG). (B) A flowchart showing how the compound screening and seed testing were performed.

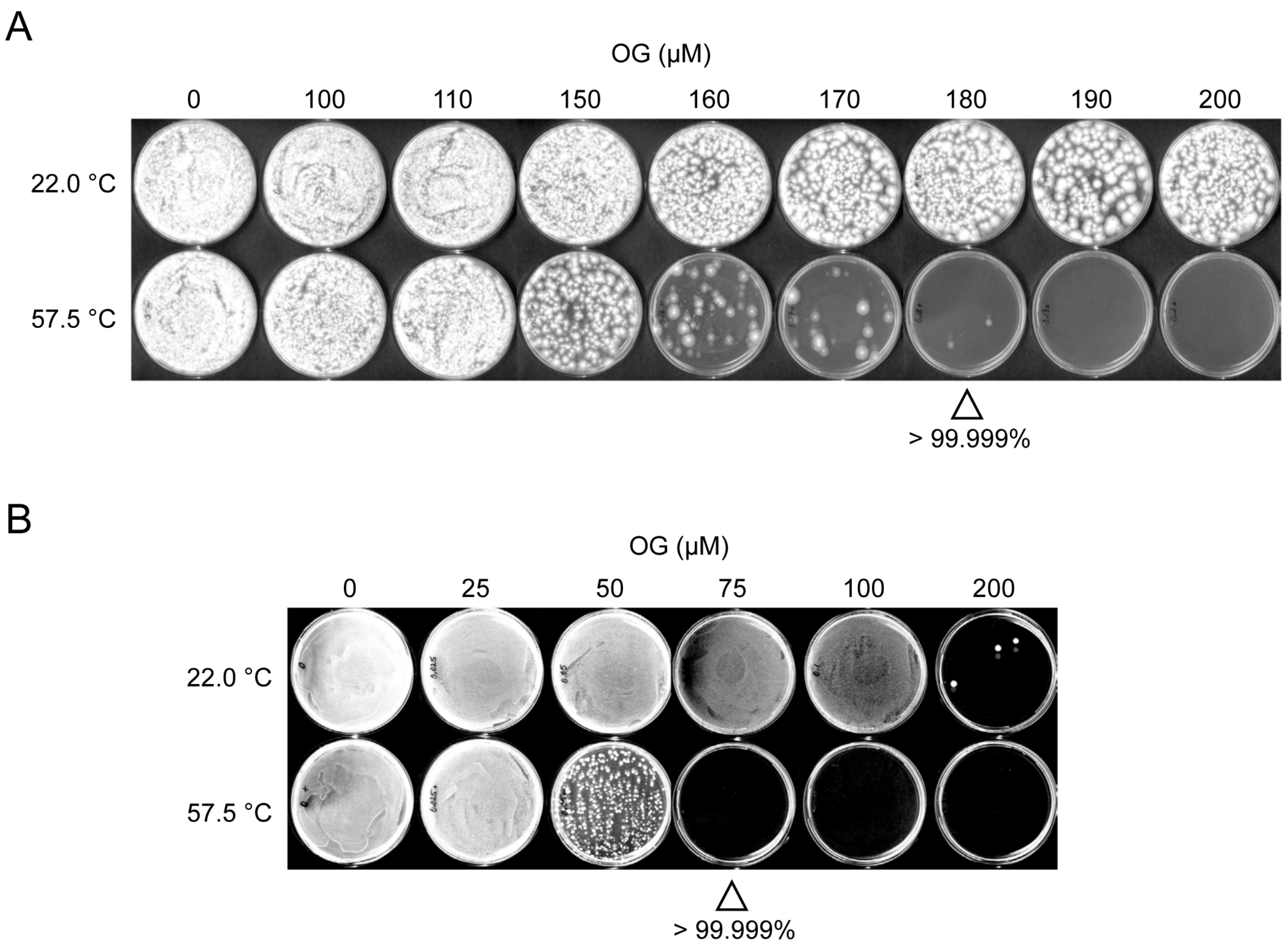

Figure S2. Heat-sensitizing capability of octyl gallate (OG) in (A) the fungus *A. flavus* strain NRRL4212 or (B) the bacterium *E. coli* strain JM109. Mild heat (57.5°C) and OG (90 sec) co-application achieved > 5 log reduction (> 99.999% microbial death) at 190 or 75 μM of OG, respectively, while independent application of each treatment, alone, resulted in the growth/survival of the microbes.

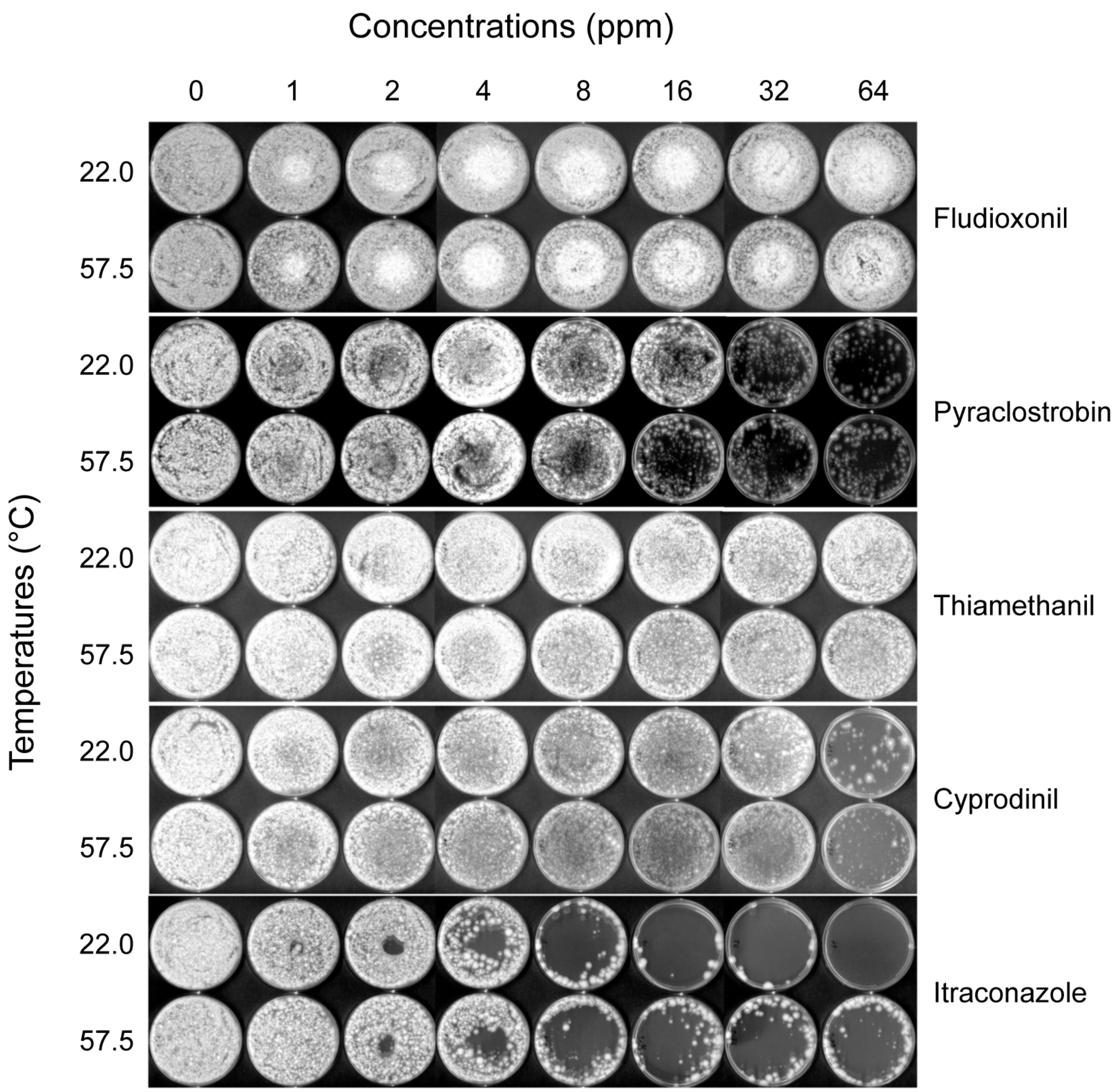

Supplementary Figure S3. Conventional drugs or fungicides do not possess heat-sensitizing activity in *A. flavus* NRRL3357. Mild heat (57.5°C) + drugs/fungicides at 64 ppm (the highest concentration applied) for 90 sec still resulted in the growth/survival of pathogens. Data shown are a representative bioassay performed using fungicides (fludioxonil, pyraclostrobin) and antifungal drugs (cyprodinil, thiamethanil, itraconazole); data not shown for caspofungin.

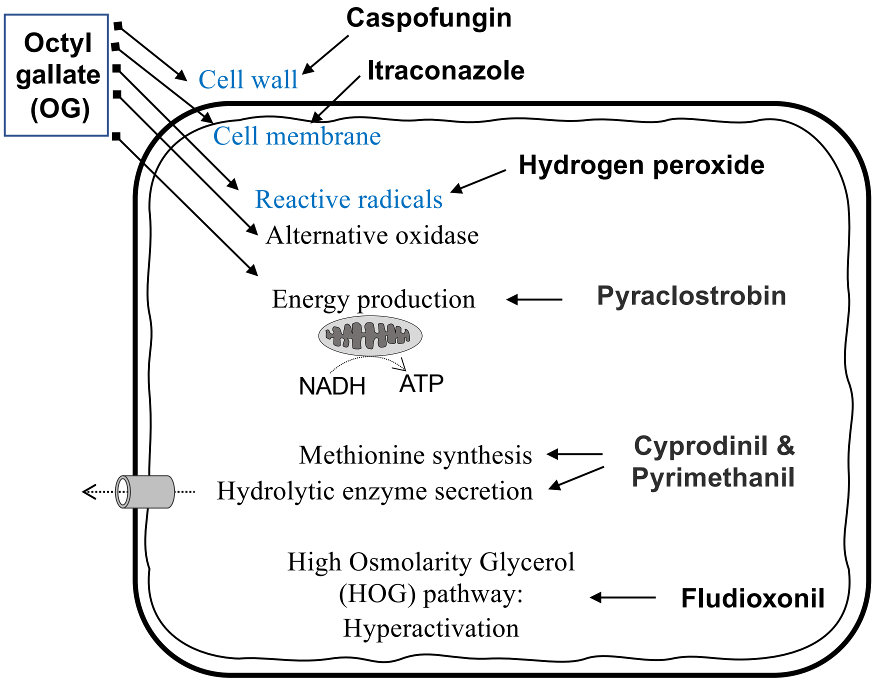

Figure S4. Summary of OG and antifungal drug targets. The cell wall, cell membrane and reactive radicals (blue characters) denote OG targets in both fungi and bacteria.

1.2 Supplementary Tables

Table S1. Microbial strains (fungi, bacteria) tested in this study.

Aspergillus	**Characteristics**	**Source/References**
A. flavus NRRL3357	Plant pathogen (aflatoxin), Human pathogen (aspergillosis), Reference aflatoxigenic strain used for genome sequencing	NRRL[a]
A. flavus NRRL4212	Plant pathogen (aflatoxin), Human pathogen (aspergillosis)	NRRL
A. parasiticus NRRL5862	Plant pathogen (aflatoxin)	NRRL
A. parasiticus NRRL2999	Plant pathogen (aflatoxin)	NRRL
A. brasiliensis ATCC16404	Environmental strain	ATCC[b]
Penicillium	**Characteristics**	**Source/References**
P. expansum W1	Plant pathogen (patulin), Parental strain	(Li and Xiao, 2008)[c]
P. expansum FR2	Plant pathogen (patulin), Fludioxonil resistant mutant derived from *P. expansum* W1	(Li and Xiao, 2008)
P. expansum W2	Plant pathogen (patulin), Parental strain	(Li and Xiao, 2008)

P. expansum FR3	Plant pathogen (patulin), Fludioxonil resistant mutant derived from *P. expansum* W2	(Li and Xiao, 2008)
P. italicum NRRL983	Plant pathogen	NRRL
P. griseofulvum NRRL2159	Plant pathogen	NRRL
P. chrysogenum NRRL824	Plant pathogen	NRRL
**Bacteria**		
Escherichia coli JM109	Model bacterium	Laboratory strain
Agrobacterium tumefaciens LBA4404	Plant pathogen	Laboratory strain

[a] NRRL, National Center for Agricultural Utilization and Research, USDA-ARS, Peoria, IL, USA.

[b] ATCC, American Type Culture Collection, Manassas, VA, USA.

[c] Li, H.X., and Xiao, C.L. (2008). Characterization of fludioxonil-resistant and pyrimethanil-resistant phenotypes of *Penicillium expansum* from apple. Phytopathology 98(4), 427-435. doi: 10.1094/phyto-98-4-0427.

Table S2. Heat-sensitizing capability of OG in bacteria.

Treatment<br><br>Bacteria	MBC of OG (μM), 22°C	MBC of OG (μM), 57.5°C
E. coli JM109	300	75
A. tumefaciens 4212	200	75
**Average**	250	75*

* The *t*-test was not performed (Duplicated samples).
